# Intersection of Brain Complexity, Functional Connectivity, and Neuropsychology: A Systematic Review

**DOI:** 10.7759/cureus.80719

**Published:** 2025-03-17

**Authors:** Rebeca de Moura Targino, Mateus Aragão A Esmeraldo, Isac Cajazeiras Falcão, Kayline Macêdo Melo

**Affiliations:** 1 Neurology and Neurosurgery, Hospital Geral de Fortaleza, Fortaleza, BRA; 2 Psychology, Universidade Federal do Ceará, Fortaleza, BRA; 3 Radiology, InRad - Instituto de Radiologia, Hospital das Clínicas da Universidade de São Paulo, São Paulo, BRA; 4 Doctors for Brazil Program, Brazil's Sistema Único de Saúde (SUS), Fortaleza, BRA; 5 Psychology, Colégio Ari de Sá, Fortaleza, BRA; 6 Psychology, Colégio Luciano Feijão, Sobral, BRA

**Keywords:** complexity, functional connectivity, functional magnetic resonance imaging, neuropsychology assessment, resting-state functional magnetic resonance imaging, systemic literature review

## Abstract

The definition of brain complexity is based on the principal property of the coexistence of a high degree of integration and differentiation within a single neural system. Despite the fruitful scope of emerging studies involving the applicability of brain complexity metrics, there is a notable scarcity of research focusing on the qualitative characteristics of conscious systems, which are recognized for their high complexity. These qualitative characteristics are expressed in complex cognitive processes, reflecting the interaction between distinct neuropsychological domains, such as attention, memory, language, and executive functions (EFs). Cognitive flexibility and inhibitory control, for instance, emerge from the dynamic integration of distributed neural networks, underscoring the interdependence between brain complexity and cognitive functioning. In light of this, the present study aimed to evaluate how studies addressing measures of functional connectivity and brain complexity, obtained through resting-state functional magnetic resonance imaging (rs-fMRI), relate to neuropsychological aspects. To achieve this, a systematic review was conducted following the Preferred Reporting Items for Systematic Reviews and Meta-Analyses (PRISMA) guidelines and based on the PICO (Patient, Intervention, Comparison, Outcome) strategy. Studies were searched in PubMed, CAPES Periodicals, and Virtual Health Library databases to identify relevant studies published between 2019 and March 2024. Articles were included based on study type, sample characteristics, methodological aspects, and specific listed variables. Exclusion criteria encompassed theoretical studies, animal research, and studies involving children/adolescents, as well as those addressing psychiatric conditions, psychoactive substance use, intervention evaluations (e.g., transcranial magnetic stimulation), and disorders of consciousness, due to limitations in applying neuropsychological measures. Possible limitations include the exclusion of studies with specific populations and clinical conditions, which may limit the generalizability of findings to broader, more diverse groups. After applying the selection criteria, 30 articles were chosen and fully analyzed. The results allowed for the establishment of characteristics of the research landscape in this area, initially highlighting a greater number of studies focused on functional connectivity compared to those directed at brain complexity. Additionally, EFs were identified as the most frequently addressed neuropsychological domain in the studies, consistent with the most commonly used evaluative measures in the research: Trail Making Test (TMT), Symbol Digit Modalities Test (SDMT), and verbal fluency tasks. The findings suggest that this is an area of study still in its early stages of development, with notable gaps in the in-depth understanding of the relationships between neural network complexity metrics and neuropsychological functioning.

## Introduction and background

Neuropsychology is based on the premise that behavior and psychological experience are expressions of the integrated and adaptive nature of the functioning brain [1]. However, a significant emerging challenge is the identification of physical properties of the brain that directly correlate with subjective experiences, a gap with substantial implications for clinical practice and contemporary ethical issues. Various theories attempt to explain brain function and its relationship to the process of human subjectivation, often serving as the foundation for the development of experimental, empirical research. One example is the study "Consciousness and Complexity," which provides a theoretical framework from which testable hypotheses about the neural substrate of conscious experience have been derived [2].

Published in 1998, the study proposes a characterization of neural processes that could explain conscious experience, highlighting two primary properties: integration and differentiation. Building on concepts such as entropy, central to understanding brain organization in terms of a physical system, the authors elaborate on a definition of complexity specifically relevant to consciousness, in which there is a coexistence of a high degree of integration and differentiation within a single system. The theoretical framework thus directs attention to a specific connectivity pattern in which researchers seek not only functionally integrated neuronal interaction networks but also signs of a highly differentiated neural system - that is, causal and efficient interactions contrasted with a high variability of non-specific mutual connections [2].

The hypothesis, theoretically formulated based on well-accepted premises in the scientific community, provided a conducive environment for the development of studies aimed at creating metrics to quantify related variables, demonstrating advances in the practical applicability of these measures. There has been significant progress in understanding the common denominators identified in studies that use complexity measures applied to brain signals, with a particular focus on consciousness, its disorders, and the potential impacts on overall psychological functioning [3].

The studies described provide significant findings regarding the logic and operationalization of strategies aimed at empirical complexity measures, while also identifying problems and limitations in this field [2,3]. Despite the fruitful scope of studies, there is a notable scarcity of research focusing on the qualitative characteristics of what are identified as neural systems with high complexity, highlighting the limitation of data that would allow for estimating correlations between complexity indices and individually manifest functionality. In this direction, the possibility arises - beyond the establishment of a general brain complexity index - to explore the contribution of specific neural networks and their idiosyncratic manifestation in terms of neuropsychological functions.

Aligned with this perspective, there is evidence of the development of approaches that allow for the investigation of the existence of patterns of functional interactions in different brain regions, providing data that contribute to the understanding of the structure-function-association paradigm concerning brain function. The notion of a brain connectivity pattern emerged from the need to explain the observation of reductions in brain activity in functional neuroimaging data when participants were in a resting state with passive visual fixation or with their eyes closed [4]. These reductions in activity were consistently observed across a wide range of experimental conditions, making the phenomenon particularly intriguing, as it was identified that they were not merely an effect of increased activity in the resting state. The presence of these reductions, thus, implied the existence of a specific pattern of brain functioning [4-6].

A significant study was conducted with the aim of mapping the functional organization of the cerebral cortex using intrinsic functional connectivity [7,8]. This approach allows for the exploration of patterns of functional interactions between different brain regions while participants are at rest or not engaged in specific tasks [9]. Using functional magnetic resonance imaging (fMRI) as the main data acquisition technique, seven main networks were identified, traditionally known as initial "parcellations." Later, recognizing the possibility of further refining the networks to capture additional nuances of brain organization, researchers developed a version in which the main networks were subdivided, resulting in 17 finer networks that allow for a more detailed representation of the complexity of functional connectivity in the human brain [7,9].

Yeo's networks provide a useful framework for understanding the brain's functional organization concerning various neuropsychological functions [10]. It is through the Visual (N1 and N2) and Somatomotor (N3 and N4) pathways, for example, that the external world can be identified, processed, and internalized individually, according to the resources available to each subject. These circuits are related to the neuropsychological domain of sensory perception, which can be conceived as the process by which the nervous system interprets and understands sensory information from the senses, resulting in a conscious perception of the surrounding environment [11].

The various forms of external stimulus processing are also associated with attentional domains, correlated with the "Dorsal Attention" (N5, N6, or DAN) and "Ventral Attention" (N7, N8, or VAN) networks. In terms of definition, attention can be situated within the notion of a set of psychological processes that enable the selection, filtering, and organization of information into controllable and meaningful units [11]. There are various components involved in the construct, but despite the multiple ways to approach the domain, there is a theoretical convergence concerning the most studied subtypes of attention, categorized in relation to the direction of consciousness, namely: (a) capacity and focus of attention/attention concentration/concentration; (b) selective attention; (c) divided attention; (d) alternating attention; (e) sustained attention; and (f) response selection and executive control [11-13].

Integrated with attentional processes is the "Executive Control Network" (N11, N12, N13, or ECN), functionally associated with what would be the basis of intentionality and the human capacity for self-management, known as executive functions (EFs) [14]. In contrast to automatic behavioral schemes, EFs enable individuals to direct their behaviors toward goals, evaluate the effectiveness and appropriateness of these behaviors, replace ineffective strategies with more efficient ones, and thus solve short-, medium-, and long-term problems [13,14].

Decision-making ability, understood within EFs, presupposes the ability to acquire, retain, and retrieve information related to previous experiences so that effective strategies for problem-solving can be selected. With this in mind, memory is emphasized as a function involved in various processes of neuropsychological functioning, playing a central role in the very definition and identity of the human being [11]. Considering the centrality of the domain, it is not difficult to grasp its association with the Default Mode Network (DMN), corresponding to networks N14, N15, and N16.

Classically considered an "intrinsic" system, the DMN comprises several cortical regions, namely the medial prefrontal cortex, posterior parietal cortex, posterior cingulate cortex, and medial temporal cortex [15], integrating external information with prior internal knowledge to construct detailed and contextualized models of situations as they unfold over time. In Alzheimer's disease, alterations in the DMN are often correlated with cognitive deficits, while in major depressive disorder, hyperactivity or hypoactivity of these regions may contribute to rumination and failures in emotional and temporal processing. The model, therefore, is directly related to neural activity that results in complex cognitive functioning, with the involvement of brain regions in memory, decision-making, imagination, and emotional processing [16].

Memory skills can be influenced by various factors, emphasizing that the formation, consolidation, retrieval, and interpretation of information are closely linked to the individual's emotional responses. Some of Yeo's networks, especially those associated with the limbic system (N9 and N10), play a crucial role in evaluating emotional stimuli, regulating emotional responses, and making emotion-based decisions. Studies on affective experiences and their anatomical and functional correlates frequently show the participation of subcortical structures (such as the amygdala, hippocampus, septal nuclei, anterior thalamic nuclei, parts of the basal nuclei, and hypothalamus) and cortical structures (such as the frontotemporal limbic cortex and cingulate gyrus) in the encoding, decoding, and recoding of emotional experiences. Furthermore, different types of emotions - such as fear, anger, sadness, and happiness - are associated with distinct patterns of brain activity in various brain regions [11,17].

The overview provided by the mapping of brain networks, as well as their correspondences in terms of neuropsychological functions, allows for an understanding of neural functioning as an integrated system, given the established correlations between different regions. The correlation between brain complexity metrics and neuropsychological functions represents an emerging field of study that can provide valuable insights into cognitive and emotional brain functioning.

Brain complexity metrics, which include measures such as entropy, fractal dimensionality, and functional connectivity, have been associated with various aspects of neuropsychological functions, including memory, attention, language, and EFs. Understanding these relationships can not only clarify the mechanisms underlying cognitive and emotional processes but also contribute to the development of new strategies for practical interventions. Therefore, this study aimed to evaluate, within the scientific literature, how studies that address measures of functional connectivity and brain complexity, obtained through resting-state functional magnetic resonance imaging (rs-fMRI), relate to neuropsychological aspects. It is considered that studies of this nature allow for a critical synthesis of the available evidence, identifying gaps in current knowledge and guiding future research in areas with a greater impact on clinical practice.

## Review

Methods

Study Type

This work is a systematic review of the literature, with a descriptive and qualitative focus, conducted in accordance with the Preferred Reporting Items for Systematic Reviews and Meta-Analyses (PRISMA) guidelines [18]. The research question that guided the search and selection of articles was formulated based on the PICO (Patient, Intervention, Comparison, Outcome) strategy and is described as follows: "How do studies that address measures of functional connectivity and brain complexity, obtained through rs-fMRI (I) in an adult population (P), relate to neuropsychological aspects? (O)."

Search Strategies and Identification

**Figure 1:**
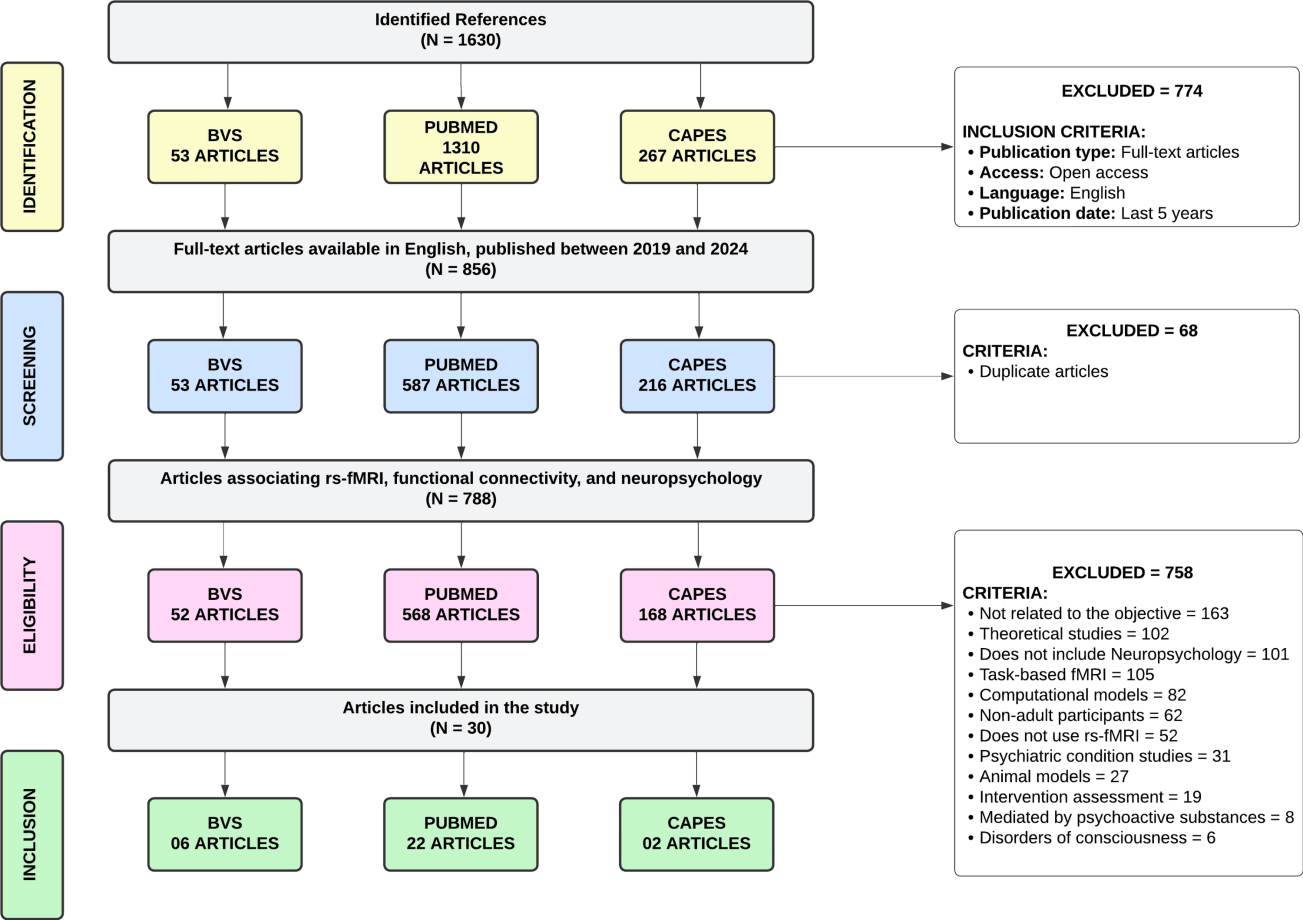
PRISMA flowchart for the search of scientific articles. rs-fMRI: Resting-State Functional Magnetic Resonance Imaging; PRISMA: Preferred Reporting Items for Systematic Reviews and Meta-Analyses

Data collection began with the search for articles published in the PubMed, CAPES Periodicals, and Virtual Health Library (VHL) databases - the latter of which aggregates several electronic databases, including MEDLINE, LILACS, and SciELO. Standardized descriptors from the Health Sciences Descriptors (DeCS/MeSH) were used, employing the following search strategy: "(Neuropsychology OR Cognitive Function) AND (Functional Connectivity OR Neural Networks) AND (Brain Complexity OR Brain Entropy) AND (Functional Magnetic Resonance Imaging OR fMRI)." The initial search, without applying filters, yielded 1,630 references, divided among different databases as shown in Figure 1.

Screening

The initial screening was based on three criteria: period, language, and file type. Complete, open-access scientific articles in English, published between 2019 and March 2024 (corresponding to the last five years), were selected. After removing duplicate files, 610 publications were included for further analysis.

Inclusion and Exclusion Criteria

The inclusion criteria encompassed primary empirical studies investigating original data, focusing on adult human participants aged 18 to 65 years. Only studies employing rs-fMRI and neuropsychological measures to assess cognitive functions were considered. Additionally, studies addressing specific neurological conditions without psychiatric comorbidities, ensuring the applicability of valid neuropsychological assessments, were included. Conversely, theoretical studies - including systematic reviews, meta-analyses, editorials, letters, and non-systematic case reports - were excluded. Studies conducted on animals, employing computational model approaches, or involving children and/or adolescents were also not considered. Furthermore, studies that did not use rs-fMRI or failed to apply valid neuropsychological measures were excluded. Research involving psychiatric conditions, psychoactive substance use, intervention evaluations such as transcranial magnetic stimulation (TMS), and disorders of consciousness was also omitted, as these conditions frequently affect neural networks, including key regions such as the DMN and the limbic system, potentially compromising the consistency of neuropsychological data interpretation. By applying these criteria, the study aimed to ensure the accuracy and relevance of the analyzed data.

The exclusion of specific populations and clinical conditions in the established exclusion criteria may affect the generalizability of the results to broader groups, presenting a potential limitation of this study. However, considering the variables to be investigated, the methodology employed is consistent with the study's objectives. After applying these criteria, 30 articles were included for full analysis.

Results

The results are derived from 30 articles. Starting from the objectives of each study, methodological aspects such as sample composition, the main neuropsychological dimensions examined, and the evaluative measures used were identified. Additionally, the primary conclusions obtained were described (Table 1).

**Table 1:** Summary of the included studies. Abbreviations: BDI-II: Beck Depression Inventory II; BTACT: Brief Test of Adult Cognition by Telephone; CESD: Center for Epidemiologic Studies Depression Scale; CVVLT: California Verbal Learning Test; DAN: Dorsal Attention Network; DKEFS: Delis-Kaplan Executive Function System; DMN: Default Mode Network; DMS-48: Delayed Matching to Sample; ECN: Executive Control Network; ESS: Epworth Sleepiness Scale; FPN: Frontoparietal Network; GNT: Graded Naming Test; HADS: Hospital Anxiety and Depression Scale; HAMA: Hamilton Anxiety Rating Scale; HVLT: Hopkins Verbal Learning Test - Revised; KBIT: Kaufman Brief Intelligence Test; MCFS: Mayo Clinic Fluctuation Scale; MMSE: Mini-Mental State Examination; Mini-SEA: Mini Social Cognition and Emotional Assessment; MoCA: Montreal Cognitive Assessment; NIHTB: National Institutes of Health Toolbox; NIHTB-CB: NIH Toolbox Cognition Battery; PAL: Paired Associates Learning Test; PASAT: Paced Auditory Serial Addition Test; POS: Personal Outcomes Scale; RAVLT: Rey Auditory Verbal Learning Test; RL/RI-16: Free and Cued Selective Reminding Test; RME: Reading the Mind in the Eyes; ROCF: Rey-Osterrieth Complex Figure; ROCFT: Rey-Osterrieth Complex Figure Test; ROT: Mental Rotation Test; rs-fMRI: Resting-State Functional Magnetic Resonance Imaging; SDMT: Symbol Digit Modalities Test; SN: Salience Network; SPAT: Spatial Memory Test 10/36; SRT: Selective Reminding Test; STAI-B: Spielberger State-Trait Anxiety Inventory; TCF: Taylor Complex Figure; TFI: Tinnitus Functional Index; TMT: Trail Making Test (Forms A and B); TTCT: Torrance Tests of Creative Thinking; VAN: Ventral Attention Network; VOSP: Visual Object and Space Perception Battery; VWM1: Verbal Working Memory Digit Span Forward Task; VWM2: Verbal Working Memory Digit Span Backward Task; WAIS-III: Wechsler Adult Intelligence Scale-Third Edition; WASI: Wechsler Abbreviated Scale of Intelligence; WCST: Wisconsin Card Sorting Test; WMS-III: Wechsler Memory Scale-Third Edition

Article	Objective	Sample Composition	Dimensions	Instruments	Conclusions
De Baene et al. (2019) [19]	Examining whether there is an association between cognitive performance and the functional network characteristics of the contralesional hemisphere in patients with glioma.	N = 45 (Male: 83% and Female: 17%). Patients eligible for tumor resection surgery for a unilateral low-grade glioma in the left hemisphere.	Verbal and visual memory; Processing and psychomotor speed; Reaction time; Complex attention; Cognitive flexibility.	Verbal Memory Test; Visual Memory Test; SDMT; Finger Tapping Test; Stroop; Continuous Performance Test; Attention Shift Test.	Functional connectivity characteristics of the contralesional hemisphere play a role in determining the severity of behavioral impairment. Better reaction time scores are achieved when the contralesional hemisphere exhibits a less segregated organization.
Loane et al. (2019)[20]	Investigate the nature of structural and functional brain abnormalities following limbic encephalitis associated with antibodies against components of the voltage-gated potassium channel complex and their role in residual memory impairment.	N = 48; Test Group = 24 (Male: 83% and Female: 17%); Control Group = 24. Patients in the post-acute phase of limbic encephalitis.	General intelligence; Semantic and episodic memory; Language; Executive functions; Visuospatial and motor function; Depression and Anxiety.	National Adult Reading Test; Warrington Recognition Memory Test; Camel and Cactus Test; GNT; WASI (Vocabulary and Similarities); WAIS-III (Digits); DKEFS; TMT (A and B); Verbal Fluency; ROCF; VOSP; ROCFT; WMS-III; HADS.	Patients showed selective memory impairment. Structural analyses revealed focal hippocampal atrophy in the medial temporal lobes, correlative atrophy in the mediodorsal thalamus, and additional volume reduction in the posteromedial cortex. No association was found between regional volumes and memory performance.
Wang et al. (2019)[21]	Demonstrate whether white matter hyperdensities, fiber integrity, and gray matter changes are associated with alterations in psychomotor processing speed.	N = 60 (Male: 50% and Female: 50%). Patients diagnosed with small vessel disease.	Neuropsychological Screening; Psychomotor processing speed.	MMSE; MoCA; TMT (A and B).	The occurrence of white matter hyperdensities, combined with the disruption of passing fiber integrity and altered functional activities in areas connected by this fiber, is associated with a decline in psychomotor processing speed.
Yu et al. (2019)[22]	Determine whether there is aberrant resting-state functional connectivity (rs-FC) between amygdala subregions and other brain areas and whether such abnormalities are related to emotional disturbances and cognitive impairment in obstructive sleep apnea.	N = 80; Test Group = 40 (Male: 100% and Female: 0%); Control Group = 40. Newly diagnosed, untreated obstructive sleep apnea patients.	Neuropsychological Screening; Degree of daytime sleepiness.	MoCA; ESS.	Obstructive sleep apnea patients showed significantly increased rs-FC between the left dorsal amygdala and the anterior lobe of the cerebellum, between the left ventrolateral amygdala, left inferior frontal gyrus, and left superior temporal gyrus, and between the right ventrolateral amygdala and the left inferior frontal gyrus. However, a significant decrease in rs-FC was observed between the right dorsal amygdala and the right prefrontal cortex in obstructive sleep apnea patients.
Campabadal et al. (2020)[23]	Characterize resting-state functional connectivity in patients with REM sleep behavior disorder using a complex network approach and determine its possible relationship with cognitive impairment.	N = 40; Test Group = 20 (Male: 70% and Female: 30%); Control Group = 20 (Male: 48% and Female: 51%). Patients with REM sleep behavior disorder.	Attention; Memory; Visuospatial domain; Executive functions; Processing speed.	WAIS-III (Digits); RAVLT; Benton Facial Recognition Test and Clock Drawing Test; Stroop Test; Phonemic Fluency; SDMT.	Compared to healthy individuals, patients with REM sleep behavior disorder exhibited reduced resting-state functional connectivity, predominantly in posterior regions. Moreover, this reduction was associated with cognitive impairment in REM sleep behavior disorder patients.
Carbó-Carreté et al. (2020)[24]	Explore the relationship that can be estimated between the functional connectivity patterns of the default mode network and Quality of Life levels in individuals with Down syndrome.	N = 44; Test Group = 22 (Male: 77% and Female: 23%); Control Group = 22. Individuals with Down syndrome.	Quality of Life	POS	The complexity indicators of connectivity networks in individuals with Down Syndrome tend to show less stable and more complex networks than those of the control group. Additionally, greater variability in these indicators was observed in the Down syndrome group. It was possible to predict the 8 dimensions of the Quality of Life model.
Chabran et al. (2020)[25]	Investigate structural and functional alterations in patients with Lewy body dementia compared to patients with Alzheimer's disease and healthy elderly individuals, and their potential links to fluctuations.	N = 184; Group 1 = 92. Patients with Lewy body dementia. Group 2 = 70. Patients with Alzheimer's disease. Control Group = 22 (Male: 50% and Female: 50%).	Memory; Executive functions; Processing speed; Instrumental Function; Social Cognition.	MMSE; RL/RI-16; DMS-48; Frontal Assessment Battery; TMT (A and B); Lexical recall; SDMT; ROCF; VOSP; Mini-SEA; RME; MCFS.	The Lewy body dementia group was characterized by decreased connectivity within the SN and attention networks, while the Alzheimer's disease group showed decreases within the SN and DMN. Additionally, higher fluctuation scores in Lewy body dementia patients were correlated with increased SN connectivity with the DAN and left thalamus, along with decreased connectivity between the SN and DMN, and between the right thalamus and both the FPN and DMN.
Hwang et al. (2020)[26]	Examine age-related processes in structural and functional neuroimaging among individuals with temporal lobe epilepsy.	N = 255; Test Group = 104 (Male: 38% and Female: 61%). Temporal lobe epilepsy patients. Control Group = 151 (Male: 42% and Female: 58%).	Executive functions; Episodic memory; Processing speed; Language.	NIHTB-CB.	The structural brain age of temporal lobe epilepsy patients was, on average, 6.6 years older, and the functional brain age was 8.3 years older than their chronological age; 16% of patients had structural brain ages above the 95th percentile of the healthy control sample, and 33% had functional brain ages above the 95th percentile.
Orwig et al. (2021)[27]	Expand research on the neural basis of creative thinking by combining weighted degree analysis of resting-state fMRI data with assessments of human and automated creativity.	N = 175 (Male: 27% and Female: 72%). Participants from the University of North Carolina at Greensboro and community members.	Divergent thinking/Creativity	Alternative Uses Task	There is lower integration of visual information during the resting state in individuals with higher scores. High local connectivity within the primary visual system represents a high degree of segregation. One interpretation of these results could be that creative people are more likely to engage in internally directed cognition (such as mind-wandering) in the absence of an external task.
Shi et al. (2020)[28]	Measure brain dynamics using entropy and examine the associations between brain entropy and divergent thinking in a large, healthy sample.	N = 386 (Male: 47% and Female: 53%). University students from Southwest University.	Divergent thinking/Creativity; Intelligence.	TTCT; Raven's Test.	Divergent thinking was consistently positively correlated with regional brain entropy in the left dorsal anterior cingulate cortex/pre-supplementary motor area and left dorsolateral prefrontal cortex. Three dimensions of divergent thinking (fluency, flexibility, and originality) were positively correlated with regional brain entropy in the left inferior frontal gyrus and left middle temporal gyrus, suggesting that more creative individuals have more flexible semantic associative networks.
He et al. (2021)[29]	Enhance understanding of the relationship between cerebral blood flow, functional networks, and neurocognition in asymptomatic adults with Moyamoya disease.	N = 35; Test Group = 15 (Male: 66% and Female: 33%). Patients with Moyamoya disease. Control Group = 20 (Male: 65% and Female: 35%).	Neuropsychological Screening; Memory; Executive functions.	Raven's Test; ROT; VWM1; VWM2; Simple Subtraction; Complex Subtraction; Word memory; Picture Memory.	Compared to controls, patients exhibited varying degrees of decline in computational ability (simple subtraction, complex subtraction, and short-term memory). Additionally, decreased cerebral blood flow was identified in the left anterior central frontal gyrus and left inferior frontal gyrus of the insular cortex with multiple network node abnormalities.
Figueroa-Jimenez et al. (2021)[30]	We analyzed how certain complexity indicators estimated in the subareas constituting the DMN could predict neuropsychological outcomes assessing Intelligence Quotient and cognitive performance in individuals with Down syndrome.	N = 64; Test Group = 32 (Male: 72% and Female: 28%). Individuals with Down syndrome. Control Group = 32.	Concept formation; Intelligence; Executive functions; Autonomy.	Frontal Assessment Battery; KBIT.	The control group exhibited lower complexity than the Down syndrome group. Additionally, the Down syndrome group showed greater variation in the distributions of complexity indicators compared to the control group. In the Down syndrome group, significant and negative relationships were found between some complexity indicators in certain DMN networks and cognitive performance scores. The Down syndrome group is characterized by more complex DMN networks and presents an inverse relationship between complexity and cognitive performance.
Foo et al. (2021)[31]	Examine age, sex, and cognitive function in association with network functional properties.	N = 17,127. Participants from the UK Biobank.	Processing speed; Memory; Executive functions; Fluid intelligence.	TMT (A and B); SDMT; Numeric memory; Pair matching.	Age was associated with a general decrease in network integration efficiency and the loss of functional specialization in specific brain regions. Sex differences were also observed, with women showing more efficient and less segregated networks compared to men.
Kadota et al. (2021)[32]	Examine whether rs-fMRI data could differentiate between progressive supranuclear palsy and multiple system atrophy through multiscale entropy analysis of BOLD signals, which estimates the complexity of temporal fluctuations in brain activity.	N = 32; Group 1 = 14 (Male: 36% and Female: 64%). Patients with progressive supranuclear palsy. Group 2 = 18 (Male: 44% and Female: 55%). Patients with multiple system atrophy.	Neuropsychological Screening; Executive functions; Depression.	MMSE; Frontal Assessment Battery; Self-Rating Depression Scale; Apathy Scale.	Patients with progressive supranuclear palsy demonstrated greater cognitive function impairments, particularly in frontal executive function. The bilateral prefrontal cortex showed lower BOLD signal entropy values across multiple time scales for progressive supranuclear palsy compared to those observed in patients with multiple system atrophy.
Omidvarnia et al. (2021)[33]	Test the hypothesis that resting-state networks exhibit unique temporal complexity fingerprints, quantified by their multiscale entropy patterns.	N = 987. Healthy young adults - Human Connectome Project.	Inhibitory control; Cognitive flexibility; Working memory; Fluid intelligence; Planning and reasoning.	Eriksen Flanker Task; WCST; N-back Task; Raven's Test; Relational Task.	The results reinforced high temporal complexity in the default mode and frontoparietal networks. Lower temporal complexity was observed in subcortical areas and the limbic system. A non-random correlation was observed between the temporal complexity of resting-state networks and fluid intelligence.
Whiteside et al. (2021)[34]	Test the hypothesis that changes in signal complexity within neural networks influence short-latency state transitions in the clinical syndromes of progressive supranuclear palsy.	N = 158; Test Group = 94. Patients with progressive supranuclear palsy. Control Group = 64.	Neuropsychological Screening	Addenbrooke's Cognitive Examination	Progressive supranuclear palsy increased the proportion of time spent in networks associated with higher cognitive functions. This effect correlated with clinical severity and a reduction in neural signal complexity.
Geng et al. (2022)[35]	Investigate the topological properties of brain functional networks in patients with isolated REM sleep behavior disorder.	N = 43; Test Group = 21 (Male: 66% and Female: 33%). Patients with isolated REM sleep behavior disorder. Control Group = 22 (Male: 41% and Female: 59%).	Neuropsychological Screening; Attention; Executive functions; Processing speed; Visuospatial skills; Verbal memory.	MMSE; TMT (A and B); SDMT; ROCFT; RAVLT.	There was a significant decrease in global network efficiency and local network efficiency in isolated REM sleep behavior disorder patients compared to the control group, while an increase in characteristic path length was identified. There was lower nodal efficiency in the occipital gyrus and lower nodal path in the frontal lobe, parietal lobe, temporal lobe, and cingulate gyrus. Functional connectivities decreased between the occipital nodes and regions where the shortest nodal path had declined. Abnormal behaviors may be associated with disrupted brain network topology and functional connectivity in isolated REM sleep behavior disorder patients.
Pini et al. (2022)[36]	Investigate the relationship between cognition and functional connectivity of major cognitive networks in Alzheimer's disease and frontotemporal dementia.	N = 65; Test Group 1 = 23. Patients with frontotemporal dementia. Test Group 2 = 22. Patients with Alzheimer's disease. Control Group = 20.	Memory; Language; Visuospatial skills; Attention.	RAVLT; PAL; Digit Span; Verbal fluency (phonemic and semantic); Token Test; TMT (A and B); ROCF; RME; Ekman 60 Faces Test.	Three principal cognitive components explained more than 80% of cognitive variance: the first component (cogPC1), memory; the second component (cogPC2), comprising emotion and language; and the third component (cogPC3), related to visuospatial skills and attentional domains. Compared to the control group, both Alzheimer's disease and frontotemporal dementia showed impairments across all cognitive domains. At the network level, the DMN showed a significant association across the group with cogPC1 and cogPC2, and the VAN with cogPC2. Conversely, the DAN and FPN exhibited a divergent pattern between diagnosis and connectivity for cogPC2.
Romanello et al. (2022)[37]	Characterize brain network organization in patients with early multiple sclerosis using time-resolved functional connectivity analysis and explore the relationship between disability, clinical outcomes across multiple domains, and altered network dynamics.	N = 202; Test Group = 101 (Male: 34% and Female: 66%). Patients with multiple sclerosis. Control Group = 101 (Male: 34% and Female: 66%).	Depression; Fatigue; Verbal learning; Memory; Visuospatial skills; Attention; Processing speed; Calculation; Verbal fluency.	Fatigue Severity Scale; BDI-II; SRT; SPAT; SDMT; PASAT; Word List Generation Test.	Connectivity alterations in early multiple sclerosis depend on network temporal dynamics and the level of disability. We revealed distinct connectivity changes between patient groups that are significant. The FPN was implicated in group functional connectivity differences and in associations with fatigue and impaired motor performance.
Wolters et al. (2022)[38]	Compare brain network organization between Parkinson’s disease subtypes (postural instability and gait disorder and tremor-dominant) and associate potential significant differences in brain connectivity with changes in cognitive performance.	N = 81; Group 1 = 54 (Male: 59% and Female: 41%). Patients with Parkinson’s postural instability and gait disorder subtype. Group 2 = 27 (Male: 78% and Female: 22%). Patients with Parkinson’s tremor-dominant subtype.	General cognitive function; Attention and Working Memory; Executive Functions; Verbal episodic memory; Language; Visuospatial Functions.	Mattis Dementia Rating Scale; SDMT; TMT (A and B); Stroop; Verbal Fluency; HVLT; Boston Naming Test; Judgment of Line Orientation Test.	The Parkinson’s postural instability and gait disorder subgroup scored worse than the Parkinson’s tremor-dominant subgroup across all cognitive domains. Resting-state fMRI network analyses suggested that the connection between the visual and sensorimotor networks is a potential differentiator between the subgroups. However, after correction for dopaminergic medication use, these results were no longer significant. There was no difference between the groups in gray matter volume.
Xu et al. (2022)[39]	Investigate whole-brain avalanche activity and its individual variability in rs-fMRI data.	N = 1200. Subjects from the Human Connectome Project.	Fluid Intelligence; Crystallized Intelligence; Working Memory.	Penn Matrix Reasoning Test Form A; NIHTB Picture Vocabulary Test; NIHTB List Sorting Working Memory Test.	Scale-free avalanche activity per subject was significantly associated with the maximum synchronization entropy of their brain activity. Meanwhile, functional connectivity complexity, as well as structure-function coupling, is maximized in subjects with maximum synchronization entropy. The neural dynamics of human participants with higher fluid intelligence and working memory scores are closer to criticality. Brain regions with dynamics close to criticality showed significant positive correlations with fluid intelligence performance, located in the prefrontal cortex and inferior parietal cortex.
Yeager et al. (2022)[40]	Test the hypothesis that the central precuneus is preferentially involved (compared to other sectors of the precuneus) in executive functions.	N = 35; Group 1 = 16 (Male: 69% and Female: 31%). Patients followed by Neurology division with lesion involving the precuneus. Group 2 = 19 (Male: 58% and Female: 42%). Patients followed by Neurosurgery division with lesion involving the precuneus.	Executive functions	TMT (A and B)	Symptom mapping revealed a statistically significant association between lesions in the central precuneus and impaired TMT performance. Additionally, a functional network derived from this region showed connectivity with other cortical areas implicated in executive function, including the dorsolateral prefrontal cortex and inferior parietal lobe.
Malotaux et al. (2023)[41]	Identify longitudinal changes in functional connectivity in patients with mild cognitive impairment characterized by β-amyloid (Aβ) status and relate these functional changes to clinical progression.	N = 60; Test Group = 44 (Male: 52% and Female: 48%). Patients with mild cognitive impairment. Control Group = 16 (Male: 50% and Female: 50%).	Memory; Language; Executive functions; Visuospatial functions.	Free and Cued Selective Reminding Test; Lexis Naming Test; Category Fluency Test; Letter Fluency Test; TMT (A and B); Luria Test; Clock Drawing Test.	The rate of changes in connectivity was significantly associated with cross-sectional and longitudinal measures of cognitive decline, but not with hippocampal atrophy. We observed greater DMN connectivity in patients with dementia compared to those with stable mild cognitive impairment in the last fMRI session. Aside from changes in DMN connectivity, no significant changes were observed within or between networks.
Parsons et al. (2023)[42]	(1) Measure processing speed in traumatic brain injury patients during acute and chronic post-injury intervals; (2) Map the anatomical distribution of edges with high structural and functional connectivity in traumatic brain injury patients; (3) Identify sub-networks with altered structural and functional connectivity bandwidth in the chronic compared to the acute post-injury interval; (4) Examine the relationship between cognitive performance and structural and functional connectivity bandwidth in each post-injury interval; (5) Examine whether changes in structural and functional connectivity bandwidth predict changes in processing speed.	N = 53 (Male: 13% and Female: 87%). Mild traumatic brain injury patients.	Verbal episodic memory; Working memory; Reasoning; Processing speed; Verbal fluency.	BTACT	Mild traumatic brain injury triggers a complex reorganization of brain connectivity from acute to chronic post-injury intervals, optimized for maximum information flow - ultimately generating increased processing speed. Our findings highlight the importance of considering indirect structural and functional connectivity to characterize neuroplasticity after trauma.
Rosemann and Rauschecker (2023)[43]	Evaluate changes in resting-state functional connectivity in tinnitus patients compared to control participants, and investigate resting-state functional connectivity in relation to patients' cognitive abilities and tinnitus distress.	N = 40; Test Group = 20 (Male: 65% and Female: 35%). Tinnitus patients. Control Group = 20 (Male: 65% and Female: 35%).	Neuropsychological Screening; Objective measurement of tinnitus independent of the presence or absence of peripheral hearing loss.	MoCA; TFI.	Positive associations with resting-state functional connectivity in the tinnitus group that were not observed in the control group. We found significant positive relationships between MoCA scores and resting-state functional connectivity of the default mode network with the left superior parietal lobule and orbitofrontal cortex; and of the precuneus with the left and right superior parietal lobule, orbitofrontal cortex, and supramarginal gyrus. We also demonstrated a significant positive correlation between TFI scores and resting-state functional connectivity of the precuneus and right lateral occipital cortex in tinnitus patients.
Simos et al. (2023)[44]	Evaluate the functional significance of aberrant connectomic patterns to explain the persistent effects of mild traumatic brain injury on emotional state and cognitive function.	N = 76; Test Group = 37 (Male: 84% and Female: 16%). Patients with chronic TBI. Control Group = 39.	Short-term and working verbal memory; Episodic memory; Visuomotor coordination speed and mental flexibility; Language; Problem-solving ability; Symptoms of depression and anxiety.	Digit Span; Greek Memory Scale; Passage Memory Subscale of the Greek Memory Scale; Taylor Complex Figures; TMT (A and B); Semantic and phonemic verbal fluency; WAIS-IV (Matrices); CESD; STAI-B.	Compared to healthy controls, the traumatic brain injury group exhibited hypoconnectivity in the temporal poles, which was positively correlated with semantic and phonemic verbal fluency, while hypoconnectivity in the right dorsal posterior cingulate region was positively correlated with the severity of depression symptoms. On the other hand, hyperconnectivity was observed in the right precentral and supramarginal gyri, which negatively correlated with semantic verbal fluency, indicating a potential ineffective compensatory mechanism.
Huang et al. (2024)[45]	Explore whether abnormal structural and functional connectivity in the acute phase could serve as indicators of longitudinal changes in imaging data and cognitive function in patients with mild traumatic brain injury.	N = 82; Test Group = 46 (Male: 41% and Female: 59%). Patients with mild traumatic brain injury. Control Group = 36 (Male: 47% and Female: 53%).	Processing speed; Sustained attention; Working memory; Motor and visual search speed; Inhibitory control.	SDMT; TMT (A and B).	In the acute phase, patients with mild traumatic brain injury demonstrated reduced structural connectivity in the dorsal attention network. Follow-up data over more than 3 months revealed signs of recovery in structural and functional connectivity, as well as cognitive function, in 22 of the 46 patients. Additionally, better cognitive function was associated with more efficient networks. Exploratory sub-network-based analysis could serve a predictive role in the prognosis of patients with mild traumatic brain injury.
Hung et al. (2024)[46]	Investigate which regions demonstrate alterations in resting-state functional connectome due to vascular cognitive impairment in hypertension using amplitude of low-frequency fluctuations, regional homogeneity, graph theoretical analysis, and network-based statistics methods.	N = 56; Test Group = 28 (Male: 64% and Female: 36%). Hypertensive patients. Control Group = 28 (Male: 25% and Female: 75%).	Episodic memory; General cognitive function; Executive functions; Mental flexibility; Processing speed; Visual scanning and search.	WAIS III (Symbol Search and Digit Span); TMT (A and B); CVVLT; Verbal Fluency Test.	In the comparison between groups of amplitude of low-frequency fluctuations and regional homogeneity, hypertension showed reduced spontaneous activity in regions corresponding to vascular or metabolic dysfunction and increased brain activity, particularly in the primary somatosensory cortex and prefrontal areas. Cognitive dysfunctions were also observed in hypertension, including executive function, processing speed, and memory. Both graph theoretical analysis and network-based statistics analyses indicated that hypertension demonstrated complex local segregation, worse global integration, and weak functional connectivity. Our findings show that resting-state functional connectivity was altered, mainly in the frontal and parietal regions, in hypertensive individuals with potential vascular cognitive impairment.
Tsai et al. (2024)[47]	Explore the variable characteristics of brain entropy over time and its potential links to general cognitive ability.	N = 396 (Male: 40% and Female: 60%).	Neuropsychological Screening; Executive functions.	MMSE; Digit Span.	The findings revealed that the global efficiency of traditional BOLD functional brain networks significantly correlated with age, with specific intrinsic functional brain networks contributing to these correlations. Additionally, local efficiency analysis demonstrated that intrinsic functional brain networks were more significant than traditional BOLD functional brain networks in identifying brain regions related to age and cognitive performance.
Xin et al. (2024)[48]	Explore the variable characteristics of brain entropy over time and its potential links to general cognitive ability.	N = 812 (Male: 49% and Female: 51%). Subjects from the Human Connectome Project.	Episodic memory; Cognitive flexibility; Inhibitory control; Fluid intelligence; Processing speed; Spatial orientation; Sustained attention; Verbal episodic memory; Working memory.	Picture Sequence Memory Test; Dimensional Change Card Sort; Eriksen Flanker Task; Penn Progressive Matrices; Pattern Completion Processing Speed; Penn Short Continuous Performance Test; Penn Word Memory Test; List Sorting.	The fractional window and mean dwell time of a brain entropy state characterized by extremely low overall brain entropy were negatively correlated with general cognitive abilities. Another brain entropy state, characterized by intermediate overall brain entropy and low brain entropy within the state localized in DMN, ECN, and part of SN, had fractional window and mean dwell time positively correlated with the aforementioned cognitive abilities. The results of our study advance our understanding of the underlying mechanisms of brain entropy dynamics and provide a potential framework for future investigations in clinical populations.

Considering the objective of this review, it was possible to identify, through descriptive statistical analysis, which neuropsychological dimensions were investigated in studies related to brain complexity/connectivity. Table 2, which depicts absolute frequency, allows for a comparative visualization of the data, showing the percentage of times each domain was cited in the analyzed articles, based on the sum of all articles (n = 30). Table 3 summarizes the main neuropsychological instruments identified, presenting, in percentage terms, the number of studies in which each instrument was used relative to the total number of articles.

**Table 2:** Domains assessed in studies on brain complexity and connectivity over the past five years: absolute frequency (n = 30).

Neuropsychological Domain	Articles Present	Frequency % (n = 30)
Executive Functions	[19,20,23,25,26,29-33,35,37,38,40-42,44-48]	n = 21 (70%)
Memory	[19,20,23,25,26,29,31,33,35-39,41,42,44-46,48]	n = 19 (63.3%)
Processing Speed	[19,21,23,25,26,31,35,37,42,45,46,48]	n = 12 (40%)
General Neuropsychological Screening	[21,22,29,32,34,35,38,43,47]	n = 9 (30%)
Attention	[19,23,35-38,45,48]	n = 8 (26.7%)
Intelligence	[20,28,30,31,33,39,48]	n = 7 (23.3%)
Visuospatial Skills	[20,23,35-38,41]	n = 7 (23.3%)
Language	[20,26,36,38,41,44]	n = 6 (20%)
Screening for Psychiatric Symptoms	[20,32,37,44]	n = 4 (13.3%)
Divergent Thinking/Creativity	[27,28]	n = 2 (6.7%)
Social Cognition	[25]	n = 1 (3.3%)
Instrumental Function	[25]	n = 1 (3.3%)
Orientation	[48]	n = 1 (3.3%)

**Table 3:** Neuropsychological instruments used in the studies. TMT: Trail Making Test; SDMT: Symbol Digit Modalities Test; MMSE: Mini-Mental State Examination; RAVLT: Rey Auditory Verbal Learning Test; MoCA: Montreal Cognitive Assessment; WAIS-III: Wechsler Adult Intelligence Scale - Third Edition; ESS: Epworth Sleepiness Scale; HADS: Hospital Anxiety and Depression Scale; WASI: Wechsler Abbreviated Scale of Intelligence; WCST: Wisconsin Card Sorting Test

Test	Articles	Articles Using the Test (n, %)
TMT (Forms A and B)	[20,21,25,31,35,36,38,40,41,44-46]	n = 12 (40%)
SDMT	[19,23,25,31,35,37,38,45]	n = 8 (26.6%)
Verbal Fluency	[20,23,25,36,38,41,44,46]	n = 8 (26.6%)
MMSE (Mini-Mental State Examination)	[21,25,32,35,47]	n = 5 (16.6%)
RAVLT (Rey Auditory Verbal Learning Test)	[23,33,35,36]	n = 4 (13.3%)
Frontal Assessment Battery	[25,30,32]	n = 3 (10%)
Digit Span	[36,44,47]	n = 3 (10%)
MoCA	[21,22,43]	n = 3 (10%)
Rey-Osterrieth Complex Figure	[20,25,36]	n = 3 (10%)
Stroop	[19,23,38]	n = 3 (10%)
WAIS-III	[20,23,46]	n = 3 (10%)
Raven's Progressive Matrices	[28,29]	n = 2 (6.6%)
Addenbrooke's Cognitive Examination	[34]	n = 1 (3.3%)
Mattis Dementia Rating Scale	[38]	n = 1 (3.3%)
ESS	[22]	n = 1 (3.3%)
HADS	[20]	n = 1 (3.3%)
Boston Naming Test	[38]	n = 1 (3.3%)
Token	[36]	n = 1 (3.3%)
WASI	[20]	n = 1 (3.3%)
WCST	[33]	n = 1 (3.3%)

Discussion

The results obtained indicate the existence of studies conducted in a wide range of contexts and with various objectives. A common denominator among these studies is their relation to neuropsychological aspects. Of the total studies, 21 articles focus primarily on the analysis of functional connectivity [19-23,25-27,29,35-38,40,42-48], while nine studies concentrate on measures of brain complexity [24,28,30-34,39,48]. Conceptually, studies focusing on connectivity primarily measure variables in terms of topological structure and function among neural networks [4-6], whereas studies using brain complexity metrics highlight variables related to brain entropy, encompassing aspects of segregation and integration within networks, along with specificity [2,3].

The scope of connectivity-focused studies can be segmented according to the different variables studied, offering an overview of the main research problems that researchers have been addressing. Notably, a significant number of studies (n = 11) investigate neuropsychological discrepancies and functional network connectivity in relation to various neurological disorders, including asymptomatic Moyamoya disease [30], chronic tinnitus [43], mild traumatic brain injury [44], idiopathic rapid eye movement (REM) sleep behavior disorder [23,35], and neurodegenerative and neurocognitive conditions such as Lewy body dementia and Alzheimer’s disease [25], Alzheimer’s disease and frontotemporal dementia [36], multiple sclerosis [37], Parkinson’s disease [38], mild cognitive impairment [41], and vascular cognitive impairment [46].

In addition to these variables, studies focusing on functional connectivity in relation to specific regions and/or structural changes were also identified. These include focal atrophy following limbic encephalitis associated with antibodies and memory alterations [20], white matter hyperdensity in small vessel disease and processing speed decline [21], amygdala subregions in obstructive sleep apnea and cognitive and emotional impairment [22], structural and functional age in temporal lobe epilepsy [26], and the central precuneus and its relation to EFs [40]. Two studies specifically examined functional and structural connectivity in traumatic brain injury, exploring its relationship with processing speed [42] and overall cognitive functions [45]. Another study utilized functional connectivity to expand knowledge of the neural basis of creative thinking [27]. Additionally, studies addressed different approaches to functional connectivity, such as interhemispheric connectivity in glioma and cognitive impairment [19], and the comparison of connectivity evaluation methods - intrinsic connectivity versus BOLD (Blood Oxygen Level Dependent) signal connectivity [47].

Regarding studies in the field of brain complexity metrics, various variables have been investigated to better understand the dynamics and functionality of neural networks in different contexts and conditions. One study examined the complexity of brain networks in individuals with Down syndrome and their association with quality of life dimensions [24]. Another study focused on the associations between brain entropy and divergent thinking, revealing how variability in brain activity can influence creativity and cognitive flexibility [28]. Furthermore, the relationship between brain complexity indicators and intelligence quotient was investigated in individuals with Down syndrome, highlighting how aspects of brain organization can predict cognitive capacity [30]. The efficiency of integration and specialization of functional networks was analyzed in relation to age, sex, and cognitive function, providing a detailed view of changes in brain connectivity [31].

The comparison of multiscale entropy in patients with progressive supranuclear palsy and multiple system atrophy revealed differences in the temporal complexity of the brain between these neurodegenerative conditions [32]. Another study focused on temporal complexity measured by multiscale entropy patterns in brain networks during the resting state, offering insight into the brain's functional organization in the absence of specific tasks [33]. Additionally, the relationship between changes in brain complexity and the rapid state transitions observed in progressive supranuclear palsy was explored, aiding in the understanding of neuronal dynamics associated with these conditions [34]. The investigation of changes in global neuronal activity and its correlation with the complexity of functional connectivity provided insights into the relationship between broad patterns of brain activity and the complexity of neural networks [39]. Finally, the analysis of the variable characteristic of brain entropy over time and its link to general cognitive ability offered a perspective on how the temporal complexity of brain activity can influence overall cognitive performance [48].

Another aspect to be noted in the results concerns the neuropsychological domains most explored in the studies, namely EFs, memory, and processing speed. The data confirm the centrality of executive circuits in overall neuropsychological functioning. EFs, which include processes such as planning, inhibitory control, cognitive flexibility, and decision-making, along with processing speed, are essential for regulating complex behaviors and adapting to new situations [11,13,14]. It is important to highlight the direct correspondence between this domain and the activation of the ECN in the brain, suggesting that EFs also play a critical role in coordinating and modulating other cognitive, affective, and behavioral functions [7,9,10,17].

In addition to executive aspects, memory also emerges as a central domain in the studies, functionally associated with the activation of the DMN. This connection is particularly relevant when considering that the DMN, which is especially active during resting states and self-referential thinking activities (such as introspection), plays a crucial role in the consolidation and retrieval of autobiographical memories, which depend on introspective processing and the integration of past experiences [15,16]. The role of the DMN in memory underscores its importance in overall cognitive functioning, as this network is involved not only in recalling past memories but also in projecting future scenarios and constructing a coherent self-narrative [11]. Furthermore, the interaction between EFs and memory suggests significant integration between the ECN and DMN, reflecting the complexity of cognitive coordination and regulation in the brain [1].

Consistent with the findings related to the neuropsychological domains most frequently addressed in the studies, the instruments identified as most commonly used are those that directly assess executive aspects of neuropsychological functioning. Among these, the Trail Making Test (TMT) stands out, evaluating skills such as cognitive flexibility and processing speed [49]; the Symbol Digit Modalities Test (SDMT) [50], which measures sustained attention and visual processing capacity; and verbal fluency tasks, which assess the ability to generate words under time constraints, reflecting the efficiency of lexical retrieval processes and executive control [51].

In addition to these specific measures, global neuropsychological screening instruments, such as the Mini-Mental State Examination (MMSE), are also prominent. The MMSE is a tool that provides a general assessment of cognitive functioning, covering different domains such as orientation, memory, attention, calculation, language, and constructive skills [52]. Its presence in the studies reflects the need for a quick and effective assessment that allows for early identification of cognitive deficits in various contexts, offering an integrated view of the overall cognitive functioning of the individuals evaluated. However, a limitation of using this and other screening instruments as the sole measure is the lack of depth they provide, which can result in a superficial assessment of certain cognitive domains. These instruments are useful for detecting warning signs but may not capture more subtle and specific aspects of neuropsychological functioning, emphasizing the importance of complementing them with more comprehensive and specific evaluations.

## Conclusions

Although research on brain complexity has advanced significantly in recent years, there remains a notable scarcity of studies that comprehensively address the relationship between brain complexity metrics and neuropsychological functions. Most existing investigations focus on isolated aspects of brain complexity, such as functional connectivity or brain entropy, without integrating these metrics into a complete framework that directly relates to cognitive and emotional processes. Furthermore, many studies use limited samples or heterogeneous methodologies, making it difficult to compare results and generalize conclusions. The lack of robust and systematic research on how brain complexity correlates with functions such as memory, attention, language, and EFs limits our understanding of the underlying mechanisms of these capacities. Future investigations should focus not only on determining whether neural networks are connected but also on understanding how these networks interact in order to identify the factors that have the greatest impact on the complexity of neuropsychological functioning. There is an urgent need for more integrated, large-scale studies that can provide a more detailed and precise view of the interactions between brain complexity and neuropsychological functions, thus contributing to the advancement of neuroscience and the improvement of clinical practices.
